# Is the dermatomal recruitment of sweating a physiological reality or a misinterpretation?

**DOI:** 10.1186/2046-7648-4-S1-A17

**Published:** 2015-09-14

**Authors:** Nigel AS Taylor, Sean R Notley, Catriona A Burdon, Elizabeth A Taylor, Norikazu Ohnishi

**Affiliations:** 1 Centre for Human and Applied Physiology, School of Medicine, University of Wollongong, Wollongong, Australia

## Introduction

A caudal-to-rostral (sympathetic dermatomal) recruitment pattern for human eccrine sweating was first described by Randall and Hertzman [1], and is widely accepted. Nevertheless, neither sudomotor activation nor sweat gland recruitment patterns were actually measured during that, or their subsequent supporting research. Instead, recruitment was derived using curves fitted to data obtained over several months, with data for separate skin regions not necessarily obtained from the same individuals. Since such data are ill-suited for drawing interpretations relative to sympathetic activation, and since Kuno [2] reported a simultaneous glandular activation across all skin regions, this hypothesis was revisited.

## Methods

Eight blindfolded, semi-nude males were exposed to a supine, resting air exposure (28 °C, 60% relative humidity). They were heated in three stages using a tube-only, water-perfusion garment: 0-5 min (water temperature 40 °C), 5-10 min (water temperature 45 °C) and beyond 10 min (water temperature 50 °C). Local sweat rates were measured using ventilated sweat capsules (3.16 cm^2^; capacitance hygrometry) positioned at four sites, none of which was covered by the garment: forehead (fifth cranial [trigeminal] nerve), dorsal hand (C6-7), lower chest (T5-6) and dorsal foot (L4-5). These positions included one cranial nerve and maximised the dermatomal range investigated. Sweat onsets were taken from the commencement of continuous local sweating, with responses tracked until steady states were achieved.

## Results

For six of these eight trials, the foot commenced sweating first, with the chest starting first on two occasions. However, during those two trials foot sweating occurred last and did not commence at all in one individual. For that person, sweat flows from the forehead and chest at the end of the trial exceeded 2 mg.cm^-2^.min^-1 ^(47.8 min). In no individual was the purported caudal-to-rostral recruitment pattern evident (foot>chest>hand>forehead). Indeed, sweating at the chest commenced last on three occasions. For each trial, the average time delays for local sweat commencement, relative to its initiation at the first site activated, were as follows: foot 3.5 min, chest 8.8 min, hand 8.1 min and forehead 9.9 min.

## Discussion

These observations are inconsistent with either a sympathetic dermatomal or a simultaneous sweat activation pattern. Randall and Hertzman [1] only presented data for two individuals displaying the former pattern, with data from another individual showing a different pattern. Subsequent self-verifying evidence also came from only two individuals [3]. On the other hand, the simultaneous activation pattern [2] was determined following visual inspection (starch iodide method), which lacks an acceptable time resolution. Thus, these scientific facts seem to have reached their expiry date and are no longer factual. Furthermore, whilst foot sweating commenced first in 75 % of the current trials, only two subjects revealed identical recruitment patterns. It appears more likely that the pattern of local sweat recruitment is an individual phenomenon.
